# The lasting effect of the Romantic view of nature: How it influences perceptions of risk and the support of symbolic actions against climate change

**DOI:** 10.1111/risa.17672

**Published:** 2024-11-03

**Authors:** Michael Siegrist, Anne Berthold

**Affiliations:** ^1^ Institute for Environmental Decisions (IED) ETH Zurich Zurich Switzerland

**Keywords:** climate change, cultural values, risk perception

## Abstract

Culture can have a major impact on how we perceive different hazards. In the Romantic period, nature was described and portrayed as mysterious and benevolent. A deep connection to nature was perceived as important. We proposed that this romantic view would be positively related to people's risk perceptions of man‐made hazards and, more specifically, to concerns about climate change. Further, we hypothesized that the Romantic perception of nature leads to a biased perception of natural hazards and that the moral component of action is of particular importance above and beyond the mere efficacy of the action. We conducted an online survey in Germany (*N *= 531), a country where Romanticism had a very widespread influence. The study shows that individuals with a Romantic view of nature perceived greater risks associated with climate change than those without such a view. In addition, those with a Romantic view of nature were more likely to support measures to reduce the risks of climate change, even when it is said that such measures are not effective. Finally, the study found a significantly higher positive correlation between Romantic views of nature and risk perceptions of man‐made versus natural hazards. The results suggest that ideas developed during the Romantic era continue to influence hazard perception in Germany.

## 1 INTRODUCTION

Understanding public risk perception is crucial because it can impact individuals’ behavior and their acceptance of technologies and regulations. Therefore, it is not surprising that a significant amount of research utilizing various methods has been conducted to examine public risk perception of different hazards (Siegrist & Arvai, 2020). In this research area, there are two distinguishable research objectives: first, examining the relevant characteristics of risks that are responsible for people being afraid of certain risks while ignoring others (Slovic, 1987) and, second, determining why people differ so much in their risk assessment of the same technology or activity (Sjöberg, 2000). The present study focuses on the latter objective. We investigated whether the Romantic period's projection of nature influences people's perceptions of nature and, consequently, their perceptions of man‐made risks today.

### 1.1 Factors influencing risk perception

It has been argued that distal factors only account for a small portion of the variance in people's risk perceptions (Sjöberg, 2003). However, this view neglects the fact that the factors that have explanatory power depend on the type of risk (e.g., smoking or pesticides) and the type of question being asked, that is, whether it pertains to the risk for the general society or personal risk, for example. Not surprisingly, it has been shown that various factors such as value orientations or psychological traits may influence risk perception among laypeople and that different predictors are important for different hazards (Siegrist & Arvai, 2020).

For some hazards, the knowledge related to a certain hazard has been shown to influence perceived risks. In the case of climate change, some fields of knowledge were positively related to concerns about climate change (Shi et al., 2015; van der Linden, 2015), but physical world knowledge was negatively related to concerns about climate change (Shi et al., 2015). For vaccination (Zingg & Siegrist, 2012) or synthetic chemicals (Saleh et al., 2019), those participants with higher levels of hazard‐specific knowledge perceived fewer risks compared with participants with less knowledge. Personality factors are another category of variables that appear to influence individuals’ risk perception. Psychological traits such as emotional stability (Chauvin et al., 2007), disgust sensitivity (Egolf et al., 2019; Scott et al., 2016), anxiety (Leikas et al., 2007), or the propensity to trust (Siegrist et al., 2021) were associated with perceived risks. The direction and magnitude of the associations depend not only on the personality trait but also on the hazard being assessed. Finally, worldviews and value orientations assist individuals in interpreting the world, and they have also been found to be correlated with risk perceptions (de Groot et al., 2013; Kahan et al., 2012; Siegrist & Bearth, 2021).

### 1.2 Cultural worldviews and human values

The present research is distinct but most closely linked to risk perception research that uses cultural worldview and values as explanatory variables. Cultural worldviews, which consist of general value orientations, help us make sense of the social world in which we are embedded, and they also influence which hazards we are concerned about (Douglas & Wildavsky, 1982). Based on this theoretical thinking, measures of worldview and cultural theory have been proposed and found to correlate with perceived risk in different domains (Johnson & Swedlow, 2021; Tumlison & Song, 2019; Xue et al., 2014). The focus of cultural worldviews is related to the social order and the way in which interactions between the state, social groups, and individuals are organized.

Cultural theory is based on the two dimensions of “grid” and “group” that result in four different cultural types (Dake, 1991; Wildavsky & Dake, 1990). The dimension “grid” is related to how much individual behavior should be regulated, and “group” is related to how much individualism or collectivism is preferred (Johnson & Swedlow, 2020). These two dimensions were used to distinguish the following four cultural types: hierarchical (high grid and high group), individualist (low grid and low group), egalitarian (low grid and high group), and fatalist (high grid and low group) (Johnson & Swedlow, 2020). These cultural values were found to influence risk perception of different hazards (Johnson & Swedlow, 2021; Kim & Jung, 2019; Marris et al., 1998; Song, 2014; Tumlison & Song, 2019).

Kahan and colleagues (Kahan et al., 2009, 2010, 2012; Kahan & Hilgard, 2016) introduced the cultural cognition theory that builds on cultural theory (Johnson & Swedlow, 2020). Cultural cognition does not distinguish between four cultural types, and the concept of fatalism is no longer considered. In this approach, the two dimensions of hierarchy versus egalitarianism and individualism versus communitarianism are distinguished. Studies using this conceptualization of cultural values have also found an association with perceived risks for various hazards (Kahan et al., 2009, 2010, 2012; Kahan & Hilgard, 2016; Parsons & Lykins, 2023; Shi et al., 2015; Siegrist & Bearth, 2021; Yang, 2016). It should also be noted that the “cultural cognitive” framework has been criticized because it is less a theory about culture and more about different preferences for the role of government in a society (van der Linden, 2016).

An even more general concept than worldviews are human values, which have been defined as the general goals that people have and that serve as guiding principles in their lives (Schwartz, 1992). This concept is even more comprehensive compared with the concept of worldviews because it goes beyond the social order. In the risk domain, studies mainly used egoistic, altruistic, and biospheric values for explaining people's risk perceptions (Cohen et al., 2020; Shi et al., 2016; Visschers et al., 2022). In the case of nuclear power, egoistic values were associated with benefit perception and altruistic values with risk perceptions, but biospheric values were not associated with the perception of nuclear power (de Groot et al., 2013). Biospheric values are related to the prevention of pollution, respecting the earth, and protecting the environment. Therefore, not surprisingly, biospheric has been positively related to people's concerns about climate change (Martin, 2023; Shi et al., 2016), and other environmental hazards (Cohen et al., 2020; Golebie et al., 2021).

### 1.3 Pro‐environmental orientation

Inspired by the environmental movements of the second half of the last century, scales have been introduced to measure participants’ pro‐environmental orientations (Dunlap et al., 2000; Dunlap & Vanliere, 1978; Thompson & Barton, 1994). These scales attempt to measure the perceived importance of environmental protection. The items are mostly in line with a utilitarian approach and do not focus on the Romantic view of nature but rather on the perceived importance of protecting the environment. Modest associations between the new environmental paradigm and risk perceptions can be observed (Sjöberg, 2000).

It should be noted that in addition to utilitarian values, intrinsic reasons for protecting nature may also be relevant (Thompson & Barton, 1994). In other words, nature should be valued and protected for its own sake. An approach that complements intrinsic and utilitarian values is called “relational values” (Arias‐Arévalo et al., 2017; Klain et al., 2017). Nature and humans are perceived as interdependent, and humans have a sense of responsibility for nature. Intrinsic and relational values are closer to the Romantic view of nature than the utilitarian approach. However, there are still differences, which will become clearer when we explain the foundations of the Romantic view. In contrast to the intrinsic and relational approaches, the Romantic approach focuses on the superiority and elevation of nature over man (Matuschek, 2024; Verwiebe & Gleis, 2024).

### 1.4 The Romantic view of nature

The current study is closely linked to research that employs worldviews and value orientations as explanatory variables for understanding individuals’ environmental risk perceptions. However, we aim to add another factor, namely a Romantic view of nature that influences people's perceptions of man‐made hazards, which is different from the worldviews and the value scales used in risk perception research. If people have a Romantic, almost mythical, view of nature as something that is always good and benevolent, this Romantic view may influence their perception of risks related to man‐made or technological hazards. More specifically, we examined whether the understanding of nature from the Romantic era has an influence on our perceptions of nature and indirectly on the perception of climate change and other man‐made risks.

The Romantic period originated at the end of the 18th century in Europe and lasted until the mid‐19th century (Matuschek, 2024). The writers of the early Romantic period were influenced by Goethe's “The Sorrows of Young Werther” (Wikipedia, 2024). In addition to the Schlegel brothers, the writers and poets Novalis, Ludwig Tieck, and E. T. A. Hoffmann are also named as central figures of early Romanticism. In England, the poets William Wordsworth and Samuel Taylor Coleridge were important representatives of Romanticism. This art epoch influenced all genres of art. In the field of painting, landscape painting experienced an upsurge during the Romantic period. Caspar David Friedrich was an important representative of Romanticism in Germany and William Turner in England.

In Romantic paintings or literature, nature is often used as an expression of inner spirituality (Matuschek, 2024). There are many facets to Romantic art. For the purposes of this study, however, we will focus primarily on how nature is depicted in paintings and described in literature, poetry, and philosophy (Görner, 2021; Illies, 2023; Matuschek, 2024; Verwiebe & Gleis, 2024). Therefore, we neglect, for example, moral beliefs, which were important for the era but not for the perception of nature.

In our paper, we will focus on Germany. The influence of Goethe's “The Sorrows of Young Werther” and the writings of the Schlegel brothers were crucial to the beginning of the Romantic era. It has been claimed that without the group around Schlegel in the German town of Jena, we would not be talking about the Romantic era (Matuschek, 2024). This era was an important period for German literature, painting, and music, and it shaped the public's perception of nature. Against this background, the study described in this paper was conducted in Germany.

The Romantic era had a major impact on perceptions of nature. In this cultural tradition, the nonhuman world is highly valued, and nature is celebrated as an antipode to technology and industrialism (Hutchings, 2007). Nature is perceived as pristine, and human influence is primarily perceived as a distortion of nature. The Romantic movement was a reaction against the Industrial Revolution and scientific reductionism in the understanding of nature. In Germany, one of the most famous painters from this era is Caspar David Friedrich (Illies, 2023; Verwiebe & Gleis, 2024), a Romantic painter who influenced the way nature is viewed and whose influence may still be shaping perceptions of natural and man‐made hazards and the acceptance of new technologies. It has been argued that Romantic ideas have influenced tourism to this day (Barsham & Hitchcock, 2013). The search for natural spectacles, such as the Alps or other landscapes, is rooted in the Romantic perception of nature. However, there can be a dark side to Romanticism. The Romantic era, with its glorification of nature, could also represent an obstacle to technological progress today and influence people's risk perceptions.

### 1.5 Measuring Romantic feelings toward nature

There are some concepts that have connections with the Romantic view of nature, such as the concept of “Living with nature” as described in the United Nations Intergovernmental Science‐Policy Platform on Biodiversity and Ecosystems (Balvanera et al., 2022) or the scales that measure intrinsic reasons for protecting nature (Thompson & Barton, 1994). However, there is no emphasis in these concepts on the superiority and elevation of nature over man. Therefore, one goal of the present study was to construct a measure that captures Romantic feelings toward nature. To construct this scale, we formulated a set of items that capture the beliefs about nature expressed by painters, poets, writers, and philosophers of the Romantic period (Görner, 2021; Illies, 2023; Matuschek, 2024; Verwiebe & Gleis, 2024).

We hypothesized that people with strong Romantic feelings about nature would perceive Romantic‐era landscape paintings as more realistic depictions of nature than people with weak Romantic feelings about nature. To test this, participants had to rate how realistic they thought Caspar David Friedrich's paintings were in their depiction of nature. We hypothesized a moderate correlation between the two measures.

### 1.6 Man‐made versus natural caused hazards

Risks caused by nature are perceived differently than those caused by humans. Experimental studies suggest that, given the same outcome, man‐made hazards are perceived more negatively than natural hazards (Rudski et al., 2011; Siegrist & Sütterlin, 2014). It has been shown that the process (i.e., human action) is more important than content in people's judgments of naturalness (Rozin, 2005). Man‐made hazards are the result of human interference with nature (e.g., genetically modified foods or synthetic fertilizers). Natural hazards, on the other hand, are not the result of human actions (e.g., lightning strikes).

It seems plausible that the Romantic view of nature is more lenient in judging natural hazards and comparatively more severe in judging man‐made hazards. Therefore, we hypothesized that there would be a strong association between the Romantic view of nature and the perceived risks of man‐made hazards. More specifically, we expected to find a higher positive correlation between Romantic perceptions of nature and the estimated risk of man‐made hazards compared to natural hazards.

Concerns about climate change are an important topic in risk perception research (Kahan et al., 2012; Shi et al., 2015). We hypothesized that having a Romantic view of nature would be positively correlated with worrying about man‐made global warming.

### 1.7 Motivating sustainable behavior and climate change concerns

Some people engage in pro‐environmental behavior to protect the environment. We hypothesized that Romantic feelings toward nature are positively correlated with such pro‐environmental behavior.

Aside from the motivation to protect the environment, adopting sustainable innovations can serve as a self‐identity or social status signal. These symbolic attributes have been shown to play an important role in pro‐environmental behavior (Noppers et al., 2014). Some studies have even suggested that signaling morally correct behavior can be more important than effective action (Sütterlin & Siegrist, 2014). We hypothesized that for people with a Romantic perception of nature, the moral component of action is of particular importance beyond the mere efficacy of action. People who adhere to the Romantic view of nature are willing to support measures that signal care for nature, even if they are ineffective. The actual effect of a behavior may, in this case, be less relevant than the intention of the behavior. We hypothesized that there is a positive correlation between Romantic perceptions of nature and support for morally correct policies even if they are ineffective.

## 2 METHODS

### 2.1 Participants

An online survey was conducted in Germany. The survey participants were recruited by a commercial provider (Bilendi GmbH) of sampling services. Participants received a small compensation. Quota sampling was used based on age (five age groups: 20–29, 30–39, 40–49, 50–59, and 60–69) and gender. Those participants who did not complete the survey or whose total survey duration was less than half of the median duration (i.e., <5 min) were excluded (*n *= 135). The final sample consisted of 531 participants. The mean age was 47 (SD = 14). In our sample, 51% were females and 49% were males.

The present study was approved by the Ethics Committee of ETH Zurich (EK No. 2023‐N‐328).

### 2.2 Questionnaire

In our online survey, the questions were asked in the order in which they are described. The order was the same for all participants; there was no randomization of item order.

First, the participants provided some sociodemographic information. They were asked about their age, gender, and education.

Based on the Romantic conception of nature, we created a 14‐item scale, as shown in Table 1. These items include the idea that nature is benevolent and inherently good and that human beings disturb the harmony and balance of nature. The mystical aspect of nature and the importance of having a deep connection with nature were also covered in some of the items. The items were formulated based on literature describing the Romantic period, focusing on the aspect of how nature was described and depicted (Görner, 2021; Matuschek, 2024; Verwiebe & Gleis, 2024). The participants responded to these statements using a response scale with six possible response options, ranging from “does not apply at all” (1) to “applies very much” (6). The Cronbach's *α* of our Romantic feeling toward nature scale was 0.93. The mean was 4.6 (SD = 0.9).

**TABLE 1:** The Romantic feeling toward nature scale.

Items	*M* (SD)
1) An intense connection with nature is important to me.	4.5 (1.3)
2) Despite scientific knowledge, the deep secrets of nature will always remain hidden from us.	4.4 (1.3
3) The harmony of nature is disturbed by humans.	4.7 (1.3)
4) Without humans, nature would be in an ideal balance.	4.7 (1.3)
5) Nothing can surpass nature in and of itself.	4.9 (1.2)
6) There is nothing more fulfilling than perceiving the beauty of nature.	4.7 (1.2)
7) Natural spectacles surpass anything mankind has ever produced.	4.8 (1.2
8) Immersing yourself in the magic of the forest is better than any medicine.	4.3 (1.4)
9) Observing a beautiful landscape fills me with a feeling of deep peace.	4.9 (1.1)
10) I find it healing when I feel part of nature.	4.5 (1.3)
11) If I had to choose between technology and nature, I would immediately opt for nature.	4.3 (1.3
12) What comes from nature cannot be bad in principle.	4.2 (1.5)
13) I care about the well‐being of nature as a whole.	4.8 (1.3)
14) I often feel a connection to the animal and plant world.	4.5 (1.3)

*Note*: *N* = 531. Responses range from 1 “does not apply at all” to 6 “applies very much.”

For measuring risk perceptions, we selected four man‐made hazards and four nature‐based hazards where human intervention is largely absent. The participants indicated for various hazards, “How high do you estimate the risk to the German population from the following hazards?” The following four hazards were used to measure perceived risks not related to human interference with nature: swim accidents, birth complications, lightning strikes, and falling during a mountain hike. The following four hazards were used to measure perceived risk in relation to man‐made hazards: preservatives, artificial sweeteners (e.g., aspartame), genetically modified food, and pesticides/chemical fertilizer. The participants responded to these statements using a response scale with six possible response options, ranging from “no risk” (1) to “very high risk” (6). Both scales—perceived risks of nonhuman and man‐made hazards—had high Cronbach's *α* with 0.85 and 0.86, respectively. The mean was 3.1 and 3.8 (SD = 1.1 and 1.1).

Concerns about climate change were measured using four items utilized in past studies (Shi et al., 2015, 2016). The items were “I worry that the state of climate is changing,” “Climate change has severe consequences for humans and nature,” “Climate protection is important for our future,” and “We must protect the climate's equilibrium.” The participants responded to these statements using a response scale with six possible response options, ranging from “does not apply at all” (1) to “applies very much” (6). This scale had a high Cronbach's *α* of 0.97. The mean was 4.9 (SD = 1.4).

Symbolic climate protection was measured using the five items shown in Table 2. The items were worded in such a way that agreeing with them implied that even if climate policies and pro‐environmental behaviors had no measurable effect, participants would still be in favor of them. These include the idea that measures should be taken to reduce CO_2_, even if they have no measurable effect on climate change, and that humans should pay for the destruction of nature. The participants responded to these statements using a response scale with six possible responses, ranging from “does not apply at all” (1) to “applies very much” (6). The Cronbach's *α* of this scale was 0.91. The mean was 3.6 (SD = 1.3).

**TABLE 2:** Items that measure symbolic climate action.

Items	*M* (SD)
1) Even if our contribution to reducing CO_2_ has no measurable effect, we are morally obliged to do so.	3.9 (1.7)
2) Avoiding consumption is important even if there is no measurable impact on the environment.	3.7 (1.6)
3) Germany should focus on CO_2_ reduction even if the amount of CO_2_ saved has no direct effect on global warming.	3.9 (1.7)
4) We should atone for our destruction of the natural world through renunciation.	3.3 (1.6)
5) It is important to set a symbol through renunciation, regardless of how the renunciation affects the environment.	3.5 (1.6)

*Note*: *N* = 531. Responses range from 1 “does not apply at all” to 6 “applies very much.”

Pro‐environmental behavior was measured with six items (Berthold et al., 2023). The participants responded to the statement, “We would like to know how willing you are to exhibit the following behaviors.” The listed behaviors were: (1.) avoiding waste (e.g., plastic); (2.) buying organic products; (3.) consuming fruit/vegetable despite stains; (4.) avoiding unnecessary driving/use public transport; (5.) avoiding unnecessary air travel; and (6.) consciously avoiding meat. The participants responded to these statements using a response scale with six possible response options, ranging from “no willingness at all” (1) to “very strong willingness” (6). The Cronbach's *α* of this scale was 0.80. The mean was 4.6 (SD = 1.0).

**FIGURE 1:**
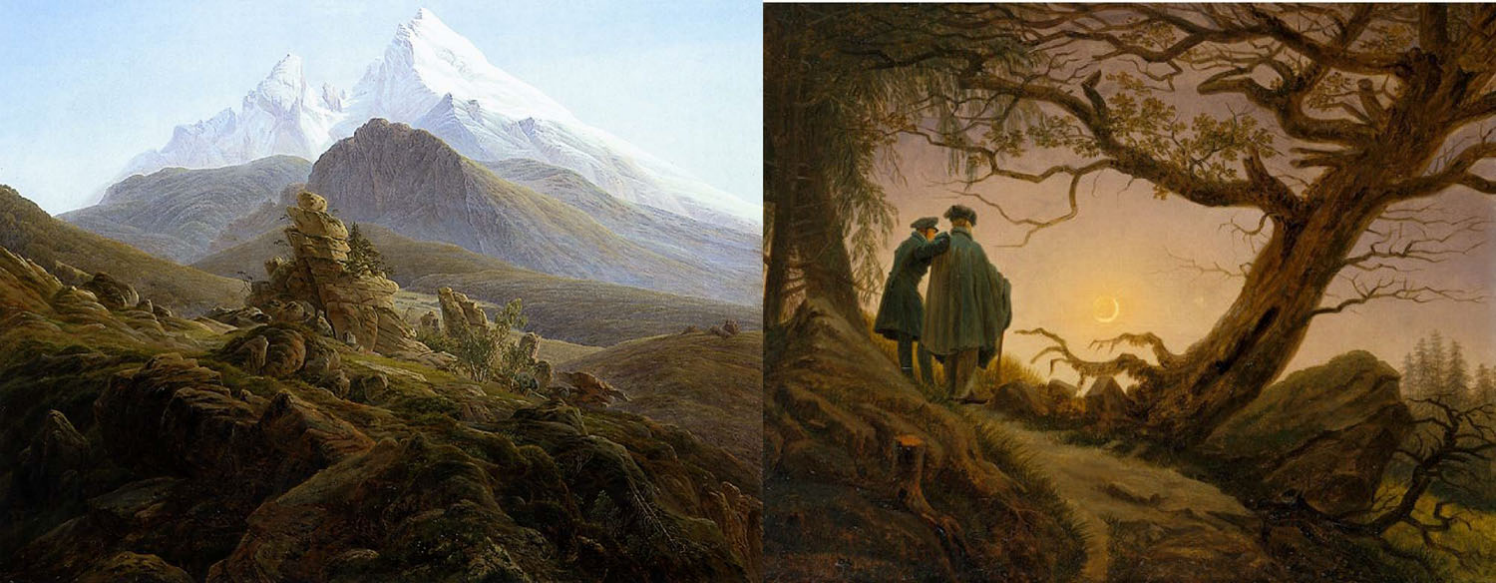
Examples of the paintings (i.e., the Watzmann, two men contemplating the moon) from Caspar David Friedrich that were used for the romantic nature picture scale. Participants had to indicate how realistic they perceived the representation of the nature to be.

The most famous German painter from the romantic era is Caspar David Friedrich (Illies, 2023; Verwiebe & Gleis, 2024). We selected five images in which humans are at best seen as observers of nature, but otherwise human activity is absent. Rather, the focus is on the mystery and beauty of nature. We also chose different types of landscapes with different colors. For the Romantic nature picture scale, the participants were asked to evaluate five of his paintings (see Figure 1). The participants responded to the question, “Does this image correspond to a realistic representation of nature?” The participants responded to this question using a response scale with six possible response options ranging from “very unrealistic” (1) to “very realistic” (6). The Cronbach's *α* of this scale was 0.81. The mean was 4.3 (SD = 1.0).

## 3 RESULTS

We conducted a linear regression analysis to examine the factors that influence the participants’ scores on the Romantic feelings toward nature scale. The regression model with the Romantic nature picture scale and the sociodemographic variables gender, age, and education was significant, *F*(4,517) = 25.76, *p* < 0.001, explaining 17% of the variance. The estimated parameters of this model are shown in Table 3. Females, older persons, and participants with lower education levels had higher values on the romantic feelings toward nature scale compared with males, younger persons, and participants with higher levels of education. Furthermore, the more the participants evaluated the paintings from the Romantic era as a realistic representation of nature, the higher their scores were on the romantic feelings toward nature scale.

**TABLE 3:** Results of a linear regression analysis with the scale Romantic feelings toward nature as the dependent variable.

	*B*	SE	*β*
Constant	3.93	0.23	
Gender	−0.36	0.07	−0.22^**^
Age	0.014	0.002	0.23^**^
Education	−0.08	0.03	−0.12^*^
Romantic nature picture scale	0.19	0.03	0.23^**^

*Note*: *N* = 521; *R* ^2 ^= 0.17. Gender has been coded as a dummy variable with females as 0 and males as 1.

*p < 0.01, **p < 0.001.

The Pearson correlations between the Romantic feelings toward nature scale, perceived risks, symbolic climate action, and pro‐environmental behavior are shown in Table 4. The Romantic feelings toward nature scale is positively correlated with perceived risks. In other words, those participants who adhere to a view of nature in the tradition of the Romantic era perceive higher risks than people who do not adhere to such a view. Furthermore, the Romantic feelings toward nature scale is strongly positively correlated with support for symbolic climate actions and willingness to engage in pro‐environmental behaviors. The participants with higher values on the Romantic feelings toward nature scale support measures fighting against climate change, even if it is clear that they are not effective.

**TABLE 4:** Pearson correlations between the Romantic nature scales, perceived risks, concern about climate change, symbolic climate action, and pro‐environmental behavior.

	1	2	3	4	5
1. Romantic feelings toward nature					
2. Perceived risks of nonhuman made hazards	0.23^**^				
3. Perceived risks of human made hazards	0.45^**^	0.43^**^			
4. Concern about climate change	0.42^**^	0.13^*^	0.20^**^		
5. Symbolic climate action	0.45^**^	0.21^**^	0.30^**^	0.71^**^	
6. Pro‐environmental behavior	0.46^**^	0.12^*^	0.23^**^	0.63^**^	0.63^**^

*Note*: *N* = 531.

*p < 0.01, **p < 0.001.

We face various hazards in our lives. Some of these are man‐made or technological hazards (e.g., genetic engineering). Other hazards may pose a threat to humans but are of natural origin (e.g., lightning). We expected higher correlations for the Romantic feelings toward nature scale and the perceived risks of the former hazards compared with the latter hazards. the observed correlations are in line with our expectations. we observed a significant Pearson correlation between Romantic feelings toward nature scale and perceived risks not related to human inferences, *r *= 0.23 (*N* = 531; *p* < 0.001; 95% confidence interval [CI] 0.15, 0.31). However, an even higher Pearson correlation was observed for human‐made hazards, *r* = 0.45 (*N* = 531; *p* < 0.001; 95% CI 0.38, 0.52). The nonoverlapping CIs indicate that the Romantic feelings toward nature scale better predicts perceived risks of human‐made hazards compared with other hazards not directly caused by humans. This significant difference was confirmed by the more formal Steiger's *Z* test, (*Z_H _* = 5.16, *p* < 0.001) (Hoerger, 2013).

**FIGURE 2:**
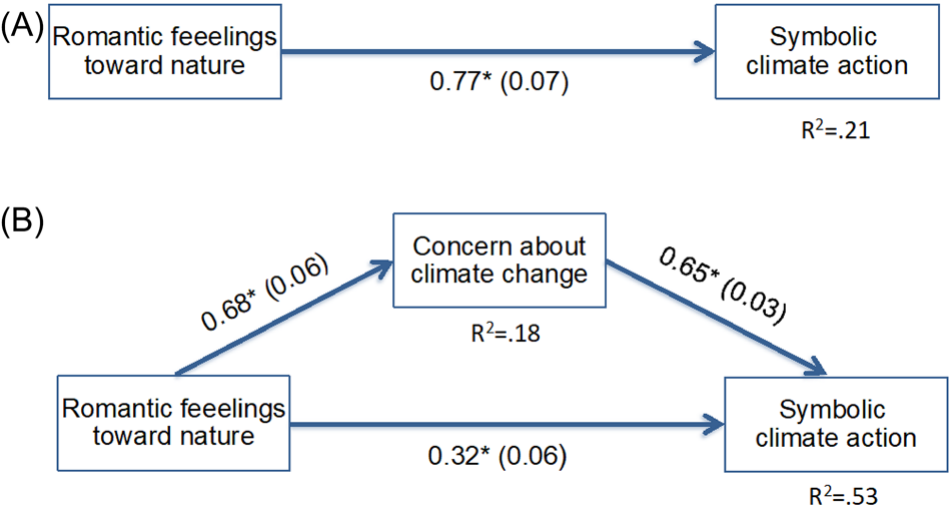
Results of the mediation analysis for the impact of Romantic feelings toward nature on the symbolic climate action. (A) the total effect of Romantic feelings toward nature on the symbolic climate action. (B) the model with concern about climate change as the mediator variable. Nonstandardized coefficients (SEs) are presented. *p < 0.001.

We investigated whether Romantic feelings about nature have only an indirect effect on symbolic climate action via concern about climate change or whether a direct effect can also be observed. This mediation effect was tested using the method proposed by Preacher and Hayes (2008). Figure 2 shows the results of the mediation analysis. Romantic feelings toward nature were positively associated with concern about climate change and had an indirect positive effect on symbolic climate action. This indirect effect is 0.44, and the bias‐corrected 95% bootstrap CI (based on 1000 bootstrap samples) suggests that this effect is significantly different from zero (95% CI: 0.35, 0.53). In addition to this indirect effect, Romantic feelings toward nature also directly influence symbolic climate action. The model explains 53% of the variance in the symbolic climate action. The results of the mediation analysis suggest that Romantic feelings about nature play an important role in the acceptance of measures against climate change that ultimately do not have a measurable effect on climate change but only indicate that the morally right thing is being done.

## 4 DISCUSSION

In the present study, we observed substantial correlations between the scale of Romantic feelings about nature and various human‐caused hazards. Our results suggest that the Romantic era may have a much longer‐lasting effect on how we perceive nature and evaluate man‐made risk than we may have thought. We introduced a new scale with items that capture the Romantic idea of nature, such as that the deep secrets of nature will always be hidden from us, that the harmony of nature is disturbed by humans, that nothing can surpass nature in and of itself, or that what comes from nature cannot be bad in principle. The items measured the extent to which people agreed with this Romantic exaggeration of nature. The Romantic perceptions of nature as mysterious and peaceful, in contrast to human activities, seemed to increase concerns about climate change and the perceived risks of various human‐made hazards.

### 4.1 Reliability and validity of the Romantic feeling toward nature scale

The scale of Romantic feelings toward nature had a very high internal consistency. As expected, we found a moderately positive association between the Romantic feelings toward nature scale and the Romantic nature pictures scale. The two measures differ not only in the type of stimuli used, verbal versus pictorial, but also in how specific they are. The items cover more different and more clearly defined concepts compared to the rating of the paintings (i.e., how realistically they depict nature). Therefore, we believe that even a modest correlation supports the validity of the Romantic feeling toward nature scale.

### 4.2 Risk perception

The Romantic perception of nature not only increases general risk perceptions, but the effects are specific. The new scale was strongly positively correlated with the perceived risks of man‐made hazards in the food domain. The participants with higher scores on the Romantic perception of nature scale perceived more risks related to food preservatives, artificial sweeteners, pesticides, or genetic engineering than the participants with lower scores on the scale. The positive correlation of the Romantic perception of nature scale with nonhuman risks (e.g., swimming accident) was significantly different from zero but also significantly smaller than the correlation of the new scale with human‐made food hazards. The finding that the Romantic perception of nature scale is more strongly correlated with anthropogenic hazards than it is with other hazards is consistent with our hypothesis. It should be noted, however, that risk perceptions of nonhuman hazards also increase with Romantic perceptions of nature. A possible reason for this could be that even for risks that are not strongly related to human associations with nature, such as swimming accidents, some human associations may be perceived by some participants (e.g., swimming accident due to misjudgment).

Climate change is a man‐made threat. Consistent with our hypothesis, we observed a strong positive correlation between Romantic feelings about nature and concern about climate change.

### 4.3 Symbolic climate action

Consistent with our expectations, we observed a positive correlation between the Romantic feelings toward nature scale and self‐reported pro‐environmental behaviors. People with stronger Romantic feelings toward nature were more likely to engage in self‐reported behaviors such as reducing waste or avoiding air travel. However, we measured self‐reported behavior. As a result, we cannot rule out the possibility that participants may have answered the items in a way that demonstrates socially desirable behavior.

The means for the items measuring symbolic climate action are close to the theoretical midpoint of the response scale used. This means that there was some disagreement among the participants regarding whether ineffective actions against climate change should be advocated for moral reasons. Our results suggest that a Romantic feeling toward nature is an important factor in why the participants perceived the moral component of action as particularly important, here beyond the mere efficacy of action. Romantic feelings not only had a direct effect but also a significant indirect effect via concern about climate change. The participants with higher scores on the Romantic feelings toward nature scale were more concerned about climate change than participants with lower scores on the Romantic feelings toward nature scale. Furthermore, both concerns about climate change and Romantic feelings toward nature had a direct effect on symbolic climate action.

### 4.4 The negative side of the Romantic view

Today, the influence of the Romantic period on public risk perception may have some undesirable side effects. The Romantic movement stood in contrast to the fundamentals of the period of enlightenment and, consequently, in contrast to a rational approach to understanding nature and the impact of technology. For laypeople, mythical thinking in the Romantic tradition may be more important for assessing the risks of modern technologies than factual/objective risk assessment (e.g., genetic engineering). This can be an important obstacle to progress if technologies are rejected based on mythical thinking rather than a rational cost‐benefit analysis.

The Romantic view of nature is related to research suggesting that in Western countries there seems to be a strong belief that nature is benevolent and inherently good (Rozin, 2005). We fully agree with Scott and Rozin (Scott & Rozin, 2020) that such a view is misguided because nature has not evolved to be good or bad for humans. Having said that, however, when human beings decide, nature is often seen as morally good and right. Identical photographs of forest ecosystem scenes described as either restored forest following natural or anthropogenic disturbance produced significantly different judgments (Campbell‐Arvai, 2019). Natural disturbances were rated much less negatively than human‐caused disturbances. As a result, restored landscapes may not be as highly valued for biodiversity by laypeople as similarly valuable pristine landscapes.

Similarly, it has been shown that people tend to be more concerned about the negative consequences of man‐made hazards than natural hazards (Siegrist & Sütterlin, 2014). A lack of perceived naturalness also seems to be an important factor in the lack of acceptance of GM (genetically modified) foods (Scott et al., 2018). Our research is closely related to this body of research, but the focus here is on individual differences, and our results also suggest that our cultural heritage may influence our perceptions. Furthermore, our research adds to the literature suggesting that people who rely on the “natural = moral” heuristic (Scott & Rozin, 2020) are prone to make biased decisions. They are more likely to support measures that are not effective on moral grounds. A Romantic view of nature could also be a challenge for structured decision‐making approaches that have been proposed to improve people's decisions (Arvai & Post, 2012).

### 4.5 How is the Romantic view related to other worldviews?

Our research adds to the evidence that, at least for some hazards, worldviews are important for a better understanding of people's risk perceptions and their acceptance of risk management measures (Dake, 1992; Douglas & Wildavsky, 1982; Earle & Cvetkovich, 1997; Kahan et al., 2009, 2010; Peters & Slovic, 1996; Shi et al., 2015; Siegrist & Bearth, 2021; Yang, 2016). We would like to stress, however, that our measure used a different approach compared with the traditional worldview measures. Both the worldviews based on cultural theory (Dake, 1992; Wildavsky & Dake, 1990) as well as the cultural cognition theory (Kahan et al., 2009, 2012) do not focus on how nature is viewed.

In the present research, we took a different approach by focusing on a specific cultural period—the Romantic period—which was very influential in Europe and shaped the perception of nature until today. We can only speculate whether Romantic feelings about nature are important in countries where no art movement has articulated this view. It may be that Romantic literature and painting simply revealed views of nature that are prevalent in other cultures and have been articulated in similar ways in other historical cultural currents. It could also be that the influence of the Romantic period is specific to certain countries. Cross‐cultural studies are needed to address this research question.

### 4.6 Limitations

Some limitations of the present study must be mentioned. Our survey used a quota sample and this sample may differ from the general population. We also cannot rule out that demand effects influenced our results, as is the case with any survey. Our aim was not to focus on all aspects of Romanticism, but on those relevant to the perception of nature. Therefore, it is possible that other aspects of Romanticism may also influence responses to perceived risk. It should be emphasized that we have examined correlations, which do not prove causality.

We had to develop new scales to measure the concepts of interest for this paper because no existing scales were available. For the Romantic nature picture scale, we used paintings from only one artist. Therefore, we cannot rule out the possibility that the specific painting style of this artist, and not the way the Romantic painter generally depicted nature, influenced our findings.

The human and nonhuman hazards used in this study differ in a number of ways. For example, the human hazards are from the food domain, whereas the nonhuman hazards are from different domains. This is a limitation, and in future studies, the hazards for the two categories should preferably be from the same domain.

### 4.7 Future research

Future research should examine how the Romantic view of nature relates to other concepts that have been found to be relevant to environmental decision‐making. It would be relevant to examine whether the Romantic view of nature leads to lower perceptions of the naturalness of GM foods and, consequently, lower acceptance of this technology. People with a view of nature strongly rooted in Romantic feelings may have protected values (Baron & Spranca, 1997) related to nature and, as a result, may be more hesitant to make trade‐offs. Investigating the relationship between protected values and Romantic views of nature would be another avenue for future research.

The present study focused on Germany. However, it should be noted that the Romantic period was not limited to Germany but was also important in other countries such as France and England. Therefore, it would be interesting to examine whether similar results could be observed in these countries. Furthermore, studies in which European culture was less important, such as in some Asian or African countries, could be used to examine the influence of Romantic feelings about nature on risk perception for countries in which the typical romantic aspect of nature has not been artistically articulated.

### 4.8 Conclusions

Our cultural traditions can have a large impact on how we perceive risk today. The results of our study are a promising first step toward demonstrating such a link. The results of this study provide some evidence that the construct of Romantic feelings toward nature is a promising explanatory variable for differences in risk perception. However, future studies are needed to investigate for which hazards this is the case and how prevalent Romantic feelings toward nature are in countries other than Germany.
